# Bacterial Contamination on Household Toys and Association with Water, Sanitation and Hygiene Conditions in Honduras

**DOI:** 10.3390/ijerph10041586

**Published:** 2013-04-18

**Authors:** Christine E. Stauber, Adam Walters, Anna M. Fabiszewski de Aceituno, Mark D. Sobsey

**Affiliations:** 1 Institute of Public Health, Georgia State University, P.O. Box 3995, Atlanta, GA 30302, USA; 2 Department of Environmental Sciences and Engineering, Gillings School of Global Public Health, University of North Carolina-Chapel Hill, Campus Box 7431, Chapel Hill, NC 27599, USA; E-Mails: adamwalters@yahoo.com (A.W.); mark_sobsey@unc.edu (M.D.S.); 3 Rollins School of Public Health, Emory University, 6000K Claudia Nance Rollins Building, 1518 Clifton Road NE, Atlanta, GA 30322, USA; E-Mail: anna.fabiszewski@emory.edu

**Keywords:** *E. coli*, toys, fomites, household water treatment

## Abstract

There is growing evidence that household water treatment interventions improve microbiological water quality and reduce diarrheal disease risk. Few studies have examined, however, the impact of water treatment interventions on household-level hygiene and sanitation. This study examined the association of four water and sanitation conditions (access to latrines, improved sanitation, improved water and the plastic biosand filter) on the levels of total coliforms and *E. coli* on existing and introduced toys during an on-going randomized controlled trial of the plastic biosand filter (plastic BSF). The following conditions were associated with decreased bacterial contamination on children’s toys: access to a latrine, access to improved sanitation and access to the plastic BSF. Overall, compared to existing toys, introduced toys had significantly lower levels of both *E. coli* and total coliforms. Results suggest that levels of fecal indicator bacteria contamination on children’s toys may be associated with access to improved water and sanitation conditions in the home. In addition, the fecal indicator bacteria levels on toys probably vary with duration in the household. Additional information on how these toys become contaminated is needed to determine the usefulness of toys as indicators or sentinels of water, sanitation and hygiene conditions, behaviors and risks.

## 1. Introduction

Lack of access to water, sanitation and hygiene is estimated to contribute more than 5% of the global burden of disease [1]. With an estimated 1.3 million child deaths due to diarrheal disease each year, there is a need to better understand the various transmission pathways of agents of diarrheal disease and identify the most impactful interventions [2]. For the past two decades, researchers have built a growing body of evidence suggesting that household-level interventions in water, sanitation and hygiene can significantly reduce diarrheal disease and improve drinking water quality [3].

Research into interventions in water, sanitation and hygiene are typically performed as randomized controlled trials (RCT) that assess the impact on diarrheal disease through the use of surveys. In addition to household surveys, these studies often employ microbiological measurements of hands and water quality parameters such as levels of residual chlorine as measures of intervention compliance or effectiveness [4,5]. Only recently have studies reported examining water and sanitation conditions and their association with household-level hygiene and sanitation, and other parameters indicative of household behaviors [6,7,8].

Pickering *et al.*, recently compared Tanzanian households using pit latrines with a concrete slab to those without the concrete slab for a number of bacterial indicators as well as some pathogens. The researchers found no differences in fecal contamination in the household environment but did find significant evidence of bacterial and pathogen contamination in soils [7]. There is a growing interest in understanding the impacts of multiple transmission pathways for microbes associated with fecal contamination, including the commonly recognized water and foodborne exposure mechanisms as well as the other less commonly measured exposures such as inanimate objects (fomites). 

In the United States, there is evidence that both porous and non-porous surfaces and objects can be vehicles of disease transmission; although much of the work has focused specifically on viruses [9,10]. Fomites, such as contaminated surfaces or objects have been shown to play an important role in the transmission of diarrhea and acute respiratory infections [9,11]. Toys, in particular, have been implicated in the spread of infection in children in healthcare settings and daycare facilities in the United States and other countries [12]. Toys have been recognized as a potential fomite for the transmission of disease in settings in developed regions, but the extent to which they may also harbor and spread infectious diseases in regions with poor access to water for hygiene and sanitation uses is poorly characterized. This study was designed to test the use of children’s toys as a measure of fecal bacterial contamination in households within an existing randomized controlled trial (water filter RCT) of a household water treatment technology, the Hydraid Biosand filter (plastic BSF) [13]. 

The BSF is an intermittently-operated slow sand filter that can produce up to 60 liters of treated water in one hour. Both the concrete and plastic BSF models have recently been shown to improve microbiological water quality in the home and reduce diarrheal disease [13,14,15]. In 2008, researchers conducted an RCT of the plastic BSF in Santa Rosa de Copan, Honduras [13]. This location was originally selected because of the lack of access to piped and/or bottled water. The water filter RCT (May 2008–February 2009) was designed to examine the ability of the plastic BSF to reduce bacterial contamination in household drinking water as well as reduce self-reported diarrheal disease compared to control households that continued normal water management practices. 

Leveraging the opportunity presented by the water filter RCT, we were able to expand data collection as a pilot study on the microbial quality of household children’s toys. The purpose of this pilot study was to examine the feasibility of collecting existing toys from households in rural Honduras as well as the utility of using introduced toys as measures of household fomite bacterial contamination. The study also examined whether or not the level of total coliforms and *Escherichia coli* found on toys in households differed with different access water and sanitation, particularly the plastic BSF as a safe water measure. The key research questions posed were:

1. What proportion of households in these communities will have an existing non-porous toy and be willing to participate?
2. What is the difference between fecal bacteria contamination levels on an existing toy having extended use and on a new, uncontaminated toy introduced for only two weeks?
3. Can we detect differences in the fecal bacteria quality of toys between households with and without access to water and sanitation improvements, such as the plastic BSF?

## 2. Experimental Section

### 2.1. Ethics Statement

The water filter RCT study protocol was approved by the Institutional Review Board of the University of North Carolina (UNC) and the Ministry of Health in Honduras in 2008 (UNC IRB # 08-0063). Informed consent for the water filter RCT was obtained during the initial household visit from the primary respondent (defined as the primary caretaker for the children and responsible for household water management practices, usually an adult female).The protocol was amended and approved for this additional pilot study on children’s toys. The primary caretaker provided informed consent to participate in the pilot study.

### 2.2. Household Recruitment and Toy Collection

During the last two months of the bi-weekly visits of the water filter RCT (January 2009), the primary caretaker was approached, told about the pilot study and invited to participate. If the primary caretaker consented, the following happened:

1. A non-porous, existing toy being used by the youngest child under five years of age in the household was collected (during household visit 1 of toy study). During this initial visit, additional households agreed to participate but were not able to provide an existing toy. They were, however, allowed to participate and provided with an introduced toy.
2. At the following visit (approximately two weeks later), the existing household toy was returned, and the toy that was introduced two weeks prior was removed for sampling. At this point in time, another toy was given to replace the introduced toy but this toy was not sampled. Toys that were collected from the household on the first visit were considered existing toys and those that were introduced into the home for two weeks were considered introduced toys.

### 2.3. Microbiological Sampling

**Figure 1:**
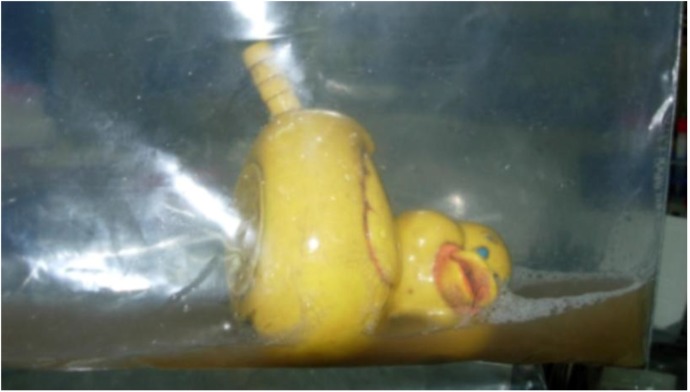
Example of sample collection bag with toy in wash solution.

Upon toy sample collection each toy was placed in a sterile sample collection bag with 115 mL of sterile wash solution (0.4 g KH_2_PO_4_, 10.1 g Na_2_HPO_4_, 1.0 mL Triton-X, 1 L water) (see Figure 1). All samples were stored at 4 °C until processed within approximately six hours of collection. Upon receipt of samples in the laboratory, each toy was massaged for 60 s in the sample collection bag and then 100 mL of the wash solution was removed. This 100 mL sample was added to a 120 mL vessel and was processed for concentrations of total coliforms and *E. coli* using the IDEXX Colilert^®^ Quanti-Tray^®^ 2000 method (IDEXX, Westbrook, ME, USA); concentrations were expressed as Most Probable Number (MPN)/100 mL.

### 2.4. Data Analysis

Bacterial concentration data from toy samples were entered into Microsoft Excel. Data from household demographic and water and sanitation data were entered into EpiInfo. Data from toy bacterial concentrations and household level data were analyzed using STATA^®^ 10 SE (Stata, StataCorp, College Station, TX, USA) and GraphPad Prism 5 (GraphPad Software Inc., La Jolla, CA, USA). Initial comparisons of prevalence of bacterial contamination for *E. coli* and total coliforms were made for existing and introduced toys using Pearson’s chi-square test. Additionally, to include concentration data for the whole range of toy samples analyzed for fecal bacteria, 0.5 MPN/100 mL was assigned to samples that fell below the lower detection limit of <1 MPN/100 mL; 4,839.2 MPN/100 mL was assigned for values that were above the detection limit of 2,419.6 MPN/100 mL. For existing toys, 23% and 47% of the samples were below the detection limit for total coliforms and *E. coli*, respectively and 43% and 16% were above the detection limit for total coliforms and *E. coli,* respectively. For newly introduced toys, 35% and 70% were below the detection limit and 20% and 6% were above the detection limit for total coliforms and *E. coli*, respectively. Data for bacteria levels on existing and introduced toys were log_10_ transformed for calculating geometric means and analysis by two sample *t*-tests, and arithmetic mean concentrations were also calculated and compared. We used two sample *t*-tests to compare differences in geometric mean concentrations of bacteria on existing and introduced toys and to determine the impact of access to water and sanitation on bacterial levels on toys. We also used the Wilcoxon Rank Sum test as a non-parametric test to examine differences between water and sanitation conditions. Data on access to water and sanitation conditions were collected from interviews performed during the water filter RCT.

## 3. Results and Discussion

### 3.1. Household Recruitment and Participation and Their Wash Status

In the water filter RCT [13], a total of 174 households were participating at the time of the pilot toy study, of which 89 households had plastic BSFs and 85 control households did not have them. All of these households were approached to participate in the study of bacterial contamination of children’s toys, and during the initial visit, a total 66 control households (78%) and 71 BSF households (80%) provided an existing toy to be sampled and gave informed consent. During the initial visit, additional households agreed to participate but were not able to provide an existing toy. They were, however, allowed to participate and provided with an introduced toy. During the second visit of the pilot study a total of 72 control households (85%) and 82 BSF households (92%) received and returned the introduced toy.

As previously reported [13], households that agreed to participate in the toy study had similar characteristics in terms of water and sanitation conditions. Most (80%) households lacked access to improved sanitation as defined by the Joint Monitoring Program of the World Health Organization and the UN. Almost 50% reported no access to a latrine on the premises and more than one third reported using unimproved sources for drinking water such as unprotected wells or springs. 

### 3.2. Bacterial Contamination on Existing and Introduced Toys

A total of 291 toy samples were collected and processed for total coliforms and *E. coli* during the two sampling periods. A total of 137 existing toys were collected and 154 toys were introduced and collected approximately two weeks later. Prevalence of *E. coli* and total coliforms on existing and introduced toys is shown in Table 1. For both *E. coli* and total coliforms, bacterial contamination was detected at statistically higher proportions on existing toys as compared to introduced toys (Pearson’s chi-square test for *E. coli p* value < 0.001, for total coliforms *p* = 0.04). Additionally, total coliforms were detected at higher proportions on both existing and introduced toys as compared to *E. coli* when prevalence was compared using Pearson’s chi-square test (*p* < 0.001 for existing and for introduce toys). 

To examine differences in concentrations of bacterial contamination, geometric mean concentrations of total coliforms and *E. coli* were compared using a two-sample *t*-test. Existing toys had significantly higher concentrations of bacterial contamination (*p* < 0.001, for *E. coli* and for total coliforms) than did introduced toys (Table 2). *E. coli* concentrations were significantly lower than total coliform concentrations for both existing toys (*p* < 0.001) and introduced toys (*p* < 0.001). In addition, arithmetic mean concentrations on existing toys were significantly higher than on introduced toys for both *E. coli* and total coliforms (*p* < 0.0001). 

**Table 1:** Prevalence of bacterial contamination on existing and introduced toys collected from households participating in this pilot study (January 2009) and an RCT of the plastic biosand filter in Copan, Honduras (May 2008–February 2009).

	Existing toy (N = 137)	Introduced toy (N = 154)	*p*-value from chi-square test ^§^
Prevalence * of *E. coli*	53%	31%	<0.001
Prevalence * of Total Coliform	77%	65%	0.04
*p*-value from chi-square test ^†^	<0.001	<0.001	

*: Detection limit was 1 MPN/100 mL wash solution. **^§^**: Pearson’s chi-square test comparing proportion of existing *vs.* introduced toys positive for bacterial contamination (*E. coli* or total coliforms). **^†^**: Pearson’s chi-square test comparing proportion of total coliforms *vs. E. coli* for toys (existing or introduced toys).

**Table 2:** Concentration of *E. coli* and total coliforms on both existing toys and toys introduced for two weeks then collected from participating households in January 2009 during an RCT of the plastic biosand filter in Copan, Honduras.

	*E. coli*		Total Coliforms	
	Existing toy (N = 137)	Introduced toy (N = 154)	Existing toy (N = 137)	Introduced toy (N = 154)
Geometric Mean (MPN/100mL)	6.4 *	1.6 *	75 *	14 *
Arithmetic Mean (MPN/100mL)	817 ^†^	347 ^†^	1126 ^†^	589 ^†^

*: Significantly different by two sample *t*-test (*p* < 0.001). **^†^**: Significantly different Wilcoxon Rank Sum test (*p* < 0.001).

Based on data collected from households on access to improved water and sanitation during the initial cross-sectional interview, further analysis was performed to determine the impact of access to these conditions on the contamination levels found on toys (both existing and introduced). The results of this analysis are presented in Table 3 for access to latrines, improved sanitation and improved water. As shown in Table 3, all of the conditions which included improved access to water and sanitation (including the presence of a latrine), demonstrated lower bacterial concentrations on both existing and introduced toys. For example, households that reported access to a latrine had a lower but not statistically significantly different geometric mean concentration of *E. coli* MPN/100 mL on existing toys compared to households without a latrine (4.6 *vs.* 9.8 respectively, *p* = 0.20 for two-sample *t*-test). This trend, while similar for all other conditions, was only statistically significant (*p* < 0.05 for two sample *t*-test or Wilcoxon Rank Sum test) for the following comparisons. Households with access to improved sanitation were found to have significantly fewer total coliforms on existing toys as compared to households that did not have access to improved sanitation (18.6 *vs.* 117.5 Total Coliforms MPN/100 mL, *p* = 0.019 for two sample *t*-test). Household with access to latrines or improved sanitation were found to have lower levels of *E. coli* on newly introduced toys as compared to households without access to those conditions. Overall, the levels of bacterial indicators on toys were the most similar when comparing those with and without improved access to drinking water. Furthermore, the differences between geometric mean concentrations of *E. coli* and total coliforms on toys for those with access to improved access to drinking water and sanitations conditions were much smaller for introduced toys. 

**Table 3:** Comparison of bacterial levels on existing toys and new toys introduced for two weeks from households with improved or unimproved water and sanitation conditions during a trial of the plastic biosand filter in Copan, Honduras.

	Latrine	No Latrine	Improved Sanitation	Lack Improved Sanitation	Improved Water	Lack Improved Water
**Existing Toys (N = 135)**	**68**	**67**	**27**	**108**	**85**	**50**
*E. coli* * (95% CI)	4.6 (2.0–10.2)	9.8 (4.1–22.8)	6.3 (1.5–27.0)	6.8 (3.5–12.9)	5.4 (2.5–11.5)	7.4 (3.0–18.4)
Total Coliforms * (95% CI)	46.8 (18.5–119.7)	141.3 (62.8–321.4)	18.6 ^†,§^ (3.8–93.3)	117.5 ^† § ^(60.3–226.9)	55.0 (24.0–123.0)	125.9 (49.4–316.3)
**Introduced Toys (N = 151)**	**73**	**78**	**29**	**122**	**95**	**56**
*E. coli* * (95% CI)	1.2 ^§^ (0.7–1.9)	2.4 ^§^ (1.2–4.7)	0.8 ^§ ^(0.4–1.6)	2.0 ^§ ^(1.2–3.3)	1.5 (0.9–2.6)	1.6 (0.8–3.3)
Total Coliforms * (95% CI)	12.9 (5.8–29.3)	16.2 (7.3–37.6)	8.9 (2.8–29.2)	16.6 (8.6–31.3)	13.5 (6.6–27.5)	15.1 (6.1–37.5)

*: Geometric mean MPN/100mL and (95% confidence interval of geometric mean). **^†^**: Significantly different by two sample t-test (*p* < 0.05). **^§^**: Significantly different by Wilcoxon Rank sum test (*p* < 0.05).

Households were also compared to determine the impact of the household water treatment device (plastic BSF) on the bacterial concentrations on children’s toys. Shown in Table 4 are the geometric mean concentrations and 95% confidence intervals for each bacterial indicator compared across toy type and the presence of the plastic BSF. The presence of the plastic BSF was associated with a statistically significant lower concentration of *E. coli* on existing toys (*p* value < 0.0097, two sample *t*-test). However, there was not statistically significant difference between *E. coli* or total coliforms levels for introduced toys (*p* = 0.38 and *p* = 0.47, respectively). In addition, while there was a difference in total coliform concentrations for existing toys (geometric mean 50 MPN/100 mL *vs.* 125 MPN/100 mL plastic BSF *vs.* control, respectively), it was not a statistically significant difference (*p* = 0.09, two sample *t*-test). Overall, existing toys had significantly higher concentrations of both total coliforms and *E. coli* than introduced toys for both plastic BSF and control households. Some water, sanitation, and hygiene conditions had significant impacts on the levels of bacterial indicators on existing household toys but not recently introduced toys. 

### 3.3. Ability to Locate and Measure Child’s Toy

This is one of the first studies to demonstrate that a child’s toy can be measured (either an existing toy or a toy newly introduced and then retrieved from the household) in studies examining the role of water, sanitation and hygiene and their impact on diarrheal disease. We found that, in the communities in the rural areas of Santa Rosa de Copan, Honduras, we were able to retrieve non-porous toys from >70% of households with children under five years of age. Furthermore, we were able to purchase, introduce and retrieve toys from households as part of a larger surveillance system of water quality and diarrheal disease for limited cost as compared to the overall cost of the water filter RCT (<5%).

**Table 4:** Comparison of geometric mean concentrations and 95% confidence intervals of *E. coli* and Total Coliforms on both existing toys and toys introduced for two weeks for households with and without the plastic BSF participating in a RCT of the plastic biosand filter in Copan, Honduras (2009).

	Plastic BSF	No Plastic BSF
**Existing Toys (N = 137)**	**N = 71**	**N = 66**
*E. coli* * (95% CI)	3.1 (1.5–6.1) ^†,§^	13.8 (5.535.3) ^†,§^
Total Coliforms * (95% CI)	45.7 (18.8–112.2)	131.8 (56.0–311.2)
**Introduced Toys (N = 154)**	**N = 82**	**N = 72**
*E. coli* * (95% CI)	2.0 (1.0–3.7)	1.3 (0.8–2.3)
Total Coliforms * (95% CI)	17.8 (8.1–38.9)	11.0 (5.0–24.5)

*: Geometric mean MPN/100 mL and (95% confidence interval of geometric mean). **^†^**: Significantly different by two sample *t*-test (*p* < 0.05). **^§^**: Significantly different by Wilcoxon Rank sum test (*p* < 0.05).

### 3.4. Levels of Bacterial Contamination on Children’s Toys

Very few studies have examined the occurrence and concentrations of bacterial indicators of fecal contamination on children’s toys in developing country settings [7,16]. In this study, we found that 53 and 77% of existing toys had detectable levels of *E. coli* and total coliforms, respectively. Geometric mean concentrations of total coliforms and *E. coli* were comparable to data from toys collected both in Peru and in a recent study in Tanzanian households which found 0.5 colony forming units of *E. coli*/100 cm^2^ surface area of fomites [7,16]. However, bacterial contamination levels found on toys in this study were often much lower than levels found on hands of both mothers and children in developing country settings [8,17]. This may be due to differences in bacteria persistence, survival and detectability on the porous and moist skin of hands *vs.* the non-porous and typically dry surface of an inanimate object such as a toy. In addition there could be key differences in activities performed that result in surface fecal contamination of either hands, inanimate household surfaces or children’s toys, as well as differences in various conditions concerning access to water, sanitation and hygiene under different environmental and behavior conditions of socio-culturally and ethnically different populations and their living conditions.

### 3.5. Water and Sanitation Factors and Association with Toy Contamination

Interestingly, for all water and sanitation conditions reported by the households (and not assigned), irrespective of whether toy was introduced or an existing toy, toys collected from households that reported improved water and sanitation conditions (including the presence of a latrine) had lower concentrations of *E. coli* and total coliforms for all improved conditions examined. While these differences in fecal bacteria levels were only statistically significant for the relationship between total coliforms and improved sanitation, this is an interesting trend that deserves further examination. Total coliforms may not be the most relevant indicator of fecal contamination in tropical settings as they have been shown to be naturally occurring in some tropical environments and may also be commonly present in soil environments in these settings [18]. Furthermore, in a recent study by Pickering *et al.*, (2012) [7], the authors found no association between households with a latrine with and without a concrete slab and household fecal contamination levels. While the relationship between improved sanitation and total coliforms was only statistically significant for three comparisons, the observed consistent trend of increased bacterial contamination on toys under conditions lack of access to water and sanitation suggests that these are issues deserving additional research. 

### 3.6. Impact of the Plastic BSF on Toy Contamination

Uniquely, this is the first study to have examined the impact of a household water treatment intervention (the plastic BSF) on bacterial levels on children’s toys. Unlike the variables previously examined with respect to access to improved water and sanitation conditions, which were not randomly assigned to households, the plastic BSF was a specific intervention randomly assigned to households. The impact of the plastic BSF on fecal bacteria levels on children’s toys varied between existing and introduced toys. Households with the plastic BSF had fewer total coliforms and *E. coli* on toys that were already present compared to households without the plastic BSF but this difference was only statistically significant for *E. coli.* In contrast, there were no such differences between plastic BSF and non-BSF households for fecal bacteria levels on new toys introduced for only a two week period. The observed differences in fecal bacteria levels between existing and new toys may be the result of the newly introduced toys being in households for only a limited amount of time (two weeks) in contrast to the existing toys which were in households for much longer time periods and were perhaps more indicative of an accumulation of bacteria levels over an extended period of time. It is also possible that the plastic BSF may not have impacted water use and hygiene behavior in the households enough to reduce levels of bacteria on fomites such as child’s toy during the two weeks that the introduced toy was left in households. It is also possible that because more than 50% of the population had access to an improved source of drinking water, the impact of the plastic BSF on water quality and hygiene, and toy contamination was reduced compared to conditions where a smaller proportion of households were using improved water. Additional geographic and hygiene status locations, where the majority of households are using untreated surface water for drinking such as in the RCT of the plastic BSF performed in Ghana would be another important setting in which to perform a similar pilot toy study [19]. 

There are some important limitations to this study of fecal contamination on children’s toys relative to water, sanitation and hygiene conditions and interventions. These limitations include the use of fecal indicator bacteria that may not always be of fecal origin or the occurrence and levels of which may not reliably indicate fecal contamination conditions, burdens or loads. Hence, there are limitations to interpreting the magnitude of fecal contamination risks from these fecal bacteria indicators. The use of bacterial indicators such as total coliforms and *E. coli* may not be representative of fecal pathogens, especially in tropical environments. Furthermore, the presence and concentrations of *E. coli* or total coliforms are not necessarily indicative of risk of exposure because other factors such as children’s hand to mouth behavior and intimate contact with the hands of child caretakers may also impact the exposure to these microorganisms. 

The bacteriological results from use of an existing toy in the household, while informative, may be difficult to interpret. Since the toy was submerged to recover its fecal microbe load, the surface area of the toy varied and could not be easily controlled for. While this recovery method allowed for more of the bacteria to be efficiently eluted off of the toy, it may make the results more difficult to compare and interpret due to differences in toy surface area and other features, such as crevasses and other surface depressions. Furthermore, we have no idea how long the existing toy had been in households, how often it may have been handled and by whom and whether or not it was cleaned or sanitized. Additionally, the introduced toy may not have been used in the same way as the existing toy, especially since household members knew that their introduced toy would be returned. Lastly, it is difficult to interpret at what points in time the target bacteria were deposited onto the toy fomite. It is possible that the bacteria were the result of a recent contamination event which could result in relatively high recovery of the organisms from the toy. Alternatively, it is also possible that the bacteria recovered from the toy represent the net yield from many events of deposition onto the surface. These organisms deposited repeatedly over time may be more difficult to recover and culture as they are more likely to have more firmly adhered to and gotten embedded in the surface matrix, making them more difficult to elute off. Furthermore, many of these bacteria may be in a viable but non-culturable physiological state. 

## 4. Conclusions

Despite study limitations, the results of this study can provide information on the potential use of sentinel objects as additional measures of water, sanitation and hygiene conditions in households. These results suggest that additional studies are needed to better understand the multiple pathways for transmission of fecal contamination, especially within the context of children’s exposures and health risks. The use of children’s toys, both existing and introduced, may be able to provide more insight into the presence and dissemination of fecal microbes and associated infectious disease risks within the household setting.
